# A minimal motif for sequence recognition by mitochondrial transcription factor A (TFAM)

**DOI:** 10.1093/nar/gkab1230

**Published:** 2021-12-20

**Authors:** Woo Suk Choi, Miguel Garcia-Diaz

**Affiliations:** Department of Pharmacological Sciences, Stony Brook University, Stony Brook, NY 11794, USA; Department of Pharmacological Sciences, Stony Brook University, Stony Brook, NY 11794, USA

## Abstract

Mitochondrial transcription factor A (TFAM) plays a critical role in mitochondrial transcription initiation and mitochondrial DNA (mtDNA) packaging. Both functions require DNA binding, but in one case TFAM must recognize a specific promoter sequence, while packaging requires coating of mtDNA by association with non sequence-specific regions. The mechanisms by which TFAM achieves both sequence-specific and non sequence-specific recognition have not yet been determined. Existing crystal structures of TFAM bound to DNA allowed us to identify two guanine-specific interactions that are established between TFAM and the bound DNA. These interactions are observed when TFAM is bound to both specific promoter sequences and non-sequence specific DNA. These interactions are established with two guanine bases separated by 10 random nucleotides (GN_10_G). Our biochemical results demonstrate that the GN_10_G consensus is essential for transcriptional initiation and contributes to facilitating TFAM binding to DNA substrates. Furthermore, we report a crystal structure of TFAM in complex with a non sequence-specific sequence containing a GN_10_G consensus. The structure reveals a unique arrangement in which TFAM bridges two DNA substrates while maintaining the GN_10_G interactions. We propose that the GN_10_G consensus is key to facilitate the interaction of TFAM with DNA.

## INTRODUCTION

Mitochondria are specialized organelles that are important for cellular ATP production and contain their own separate genome. The mitochondrial DNA (mtDNA) encodes 37 genes. Thirteen are translated into protein subunits of the electron transport chain (ETC) on the mitochondrial inner membrane, two are the RNA components of the mitochondrial ribosome and the rest are tRNAs essential for mitochondrial translation (1). The 37 genes are unequally distributed on two DNA strands, named light and heavy owing to their different buoyant density (2), and expressed from three promoters: the light strand promoter (LSP), and the heavy strand promoters 1 and 2 (HSP1 and HSP2) (3,4). Although the presence of HSP2 has been suggested by various studies (5–9), doubts still exist as to its relevance *in vivo* (5). LSP and HSP1 are located closely in the genome but not overlapping, and are responsible for transcription of opposite strands of the mtDNA.

Mitochondrial transcription is initiated by three nuclear-encoded proteins: mitochondrial transcription factor A (TFAM), mitochondrial transcription factor B2 (TFB2M) and the mitochondrial RNA polymerase (POLRMT). The initiation machinery recognizes the promoters and unwinds the DNA double-helix for mRNA synthesis. POLRMT is then able to synthesize RNA, but *in vitro* evidence suggests that it can only synthesize short RNA transcripts (10). An elongation factor, TEFM, is required for increased processivity, which is essential for the transcription machinery to reach the termination signals and to overcome early transcription termination at CSB (conserved sequence block) sites (10–14). Structures of transcription initiation and elongation complexes have brought significant insight into the mechanisms of transcription (14–17).

Human TFAM is a nuclear-encoded protein composed of 246 residues. It contains an N-terminal 42-aa signal sequence required for translocation into mitochondria, which is then proteolytically cleaved upon import to generate the mature form of TFAM (43–246) in the mitochondrial matrix (18). TFAM is composed of two tandem high-mobility group (HMG) domains connected by an α-helix linker. The high binding affinity of TFAM to DNA is mediated through the HMG domains with the aid of an α-helix linker and a C-terminal tail (3TMM and 3TQ6) (19,20). TFAM plays a key role in transcription initiation. It recognizes the promoter sites and recruits other initiation factors (21–24). Key to this function is TFAM’s ability to specifically bind to the mitochondrial promoters. The TFAM binding site in LSP has been well characterized, as TFAM binding results in a very clear 23 nucleotide footprint (21,22). However, the DNaseI footprint at HSP1 is diffuse (21). Furthermore, the presence of varying TFAM concentrations affects LSP and HSP1 initiation differentially (25,26), indicating that transcription initiation at both promoters might be differentially regulated (26).

Intriguingly, TFAM plays an additional function as an mtDNA packaging factor: TFAM can coat the mtDNA and package it in a condensed form, helping to structure the mitochondrial nucleoid (27–29). Importantly, this TFAM function requires non sequence-specific binding throughout the mtDNA genome. Although TFAM crystal structures in complex with both specific and non sequence-specific sequences have been determined (19,20,30), how TFAM differentiates between both types of sequences remains unclear. It has been suggested that the TFAM C-terminal tail may play a major role in specific sequence recognition (31), but evidence for this has not been observed in any of the crystal structures since the C-terminal tail was not fully resolved (19,20,30). In the structures, TFAM residues mostly make non sequence-specific contacts with the phosphate groups and sugar rings of the DNA substrates. However, a few residues interact with the nucleic acid bases through hydrogen bonds, which likely contributes to the specific binding of TFAM on promoters (19,20,30). Furthermore, it has been reported that DNA kinks and hydrophobic interactions induced by HMG boxes can confer sequence-specificity to the interaction (32–34).

Here, we have determined additional crystal structures of TFAM in complex with LSP (LSP_B) and a non-specific (NS2) DNA substrate. Through structural analysis, we have identified a guanine-specific consensus that might facilitate sequence recognition by TFAM. The consensus is composed of two guanine bases separated by 10 variable nucleotides (GN_10_G). Strikingly, this structural feature has been observed in all human TFAM crystal structures determined to date (19,20,30,35). Together with the crystal structures, we performed biochemical assays to provide evidence that TFAM preferentially binds to this GN_10_G consensus, and that this interaction might play a role in mitochondrial transcription. In addition, our structures reveal a unique DNA binding mode whereby a single TFAM molecule is able to bridge two DNA fragments. Our structural and biochemical results provide a mechanistic clue to understand how TFAM recognizes its substrates to carry out its diverse functions on mtDNA.

## MATERIALS AND METHODS

### Protein expression and purification

TFAM was cloned into a modified pET22 vector using EcoRI and XhoI. The vector harbored an N-terminal histidine-tagged maltose binding protein (MBP) cleavable by TEV protease. The resulting plasmid was transformed into ArcticExpress (DE3), and cells were grown in YB medium at 37°C until OD_600_ reached ∼0.8. The expression of TFAM (aa 43–246) was induced by the addition of 0.3 mM IPTG at 16°C for 15 h. The cells were pelleted by centrifugation at 6500 rpm for 15 min, and lysed in lysis buffer (20 mM HEPES pH 8.0, 1 M KCl, 20 mM Imidazole) by sonication. The lysate was cleared by centrifugation at 13 000 rpm for 1 h, and the supernatant was applied to a Ni-NTA column equilibrated in the lysis buffer. The sample was eluted using lysis buffer containing 500 mM Imidazole. The histidine-tagged MBP was cleaved by TEV at 4°C overnight. TFAM was further purified using HiTrap Heparin HP (GE healthcare) equilibrated in 20 mM HEPES pH 8.0 and 1 mM DTT, and eluted by a linear KCl gradient from 0 to 1000 mM. As a final step, TFAM was applied to a Superdex 200 16/600 GL column (GE healthcare) equilibrated in 20 mM HEPES pH 8.0, 150 mM KCl and 1 mM DTT. The TFAM peaks were pooled and concentrated using an Amicon Ultra-15 concentrator (Millipore, 10k cutoff) to ∼15 mg/ml and stored at −80°C. The TFAM mutant, S61A, was generated by site-directed mutagenesis, and purified using the same procedure.

### Crystallization

All oligos were synthesized using an automated DNA synthesizer and purified by HPLC and ethanol precipitation (36). For the crystallization of TFAM with NS2, TFAM (410 μM) was mixed with 615 μM annealed NS2 (1:1.5 molar ratio of Protein:DNA), and incubated on ice for 30 min. Crystals were grown using the hanging drop vapor diffusion method. 1 μl of protein complex was mixed with an equal volume (1 μl) of each well solution. The TFAM-NS2 crystal was grown in 0.1 M Bis–Tris pH 6.5 and 24% PEG 2000 MME. The crystallization of TFAM-LSP_B was performed by the same procedure, and the crystal was grown in 0.1M HEPES pH 7.5, 0.05 M MgCl_2_ and 32.5% PEG MME 550. TFAM-LSP_B and -NS2 crystals were cryo-protected in their respective buffers (NS2: 0.1 M Bis–Tris pH 6.5, 30% PEG 2000 MME, and 20% ethylene glycol, LSP_B: 0.1 M HEPES pH 7.5, 0.05 M MgCl_2_ and 36% PEG MME 550), and cryocooled in liquid nitrogen.

### Data collection and structure determination

Data were collected at beamlines X25 (NS2) and X29 (LSP_B) of the National Laboratory Synchrotron Light Source (NSLS) at Brookhaven National Laboratory. The data were processed and scaled using XDS (37) and AIMLESS (38) in the autoPROC pipeline (NS2) (39). Both structures were phased by molecular replacement using Phaser (40). The resulting models were manually built using COOT (41), and the structures were refined using Phenix (42) and BUSTER (43). All structural figures were prepared using PYMOL (www.pymol.org).

### *In vitro* transcription initiation assay

Both LSP (171-470) and HSP (491-790) were cloned with NcoI and HindIII into the pET-22 vector. For the run-off transcription assay, the LSP or HSP vector was linearized using NcoI or HindIII, respectively. All transcription factors were pre-mixed as a 1:1:1 molar ratio of TFAM: TFB2m: POLRMT. Each reaction mixture (20 μl) contained transcription buffer (20 mM HEPES pH 8.0, 40 mM KCl, 5 mM DTT, 1 mM EDTA and 10 mM MgCl_2_), 20 ng of linearized LSP or HSP, 0.02 μM protein mixture, 0.3 μCi [P^32^]-αUTP and 3 μl rNTP mixture (0.4 mM ATP, 0.15 mM CTP and GTP, 0.01 mM UTP). The mixture was incubated at 32°C for 30 min, and then terminated by the addition of an equal volume of 20 mM EDTA, 1% SDS, 300 mM sodium acetate and 20 μg calf thymus DNA. The transcription product was ethanol-precipitated and resuspended in 20 μl of loading buffer. The samples were resolved on 10% TBE-Urea gel. The gel was dried for 2 h and exposed to a phosphor screen (GE Healthcare) overnight. The transcription products were visualized using a Typhoon 9000 and analyzed using ImageQuant (GE healthcare). The quantified data were presented as mean ± SEM (standard error of the mean) from three independent experiments. The statistical significance was calculated with a two-tailed unpaired *t* test.

### Electrophoretic mobility shift assay (EMSA)

The 28-bp nonspecific oligonucleotide (DNA^GG^) was chosen from the human mtDNA genome (6694–6721; within the cytochrome C oxidase subunit I gene), containing a GN_10_G consensus in the middle. In DNA^AA^, both guanines were replaced to adenines. Both substrates were labeled by a Cy3 fluorophore at their 5′-end, and the following complementary sequences were annealed: DNA^GG^: 5′-/Cy3/AAAAAGAA**C**CATTTGGATA**C**ATAGGTAT-3′; 5′-ATACCTAT**G**TATCCAAATG**G**TTCTTTTT-3′ and DNA^AA^: 5′-/Cy3/AAAAAGAA**T**CATTTGGATA**T**ATAGGTAT-3′; 5′-ATACCTAT**A**TATCCAAATG**A**TTCTTTTT-3′. The underlined bold letters on DNA^GG^ and DNA^AA^ represent the GN_10_G consensus and the altered one, respectively. In order to reduce nonspecific interactions between TFAM and DNA, the NaCl concentration was optimized from 0 to 600 mM, and decided as 500 mM due to the initial appearance of free DNA at this concentration. For apparent *K*_D_ calculations, each reaction mixture (20μl) was composed of a binding buffer (10 mM HEPES pH 8.0, 2 mM DTT, 130 μg/ml BSA, 500 mM NaCl, 5% glycerol), 100 nM DNA substrate, and 0–600 nM TFAM. The reaction mixtures were incubated on ice for 30 min and at room temperature for 10 min. The mixtures were then mixed with the equal volume (20 μl) of a loading buffer (5% glycerol and Tris/borate/EDTA buffer) and loaded on 6% non-denaturing polyacrylamide gels. The results were recorded using Typhoon 9000 and analyzed using ImageQuant (GE healthcare). *K*_D_ was calculated using the following saturation binding equation:

$$\begin{equation*}{{{{Y }} = {\rm{ }}{{{B}}_{{\rm{max}}}}*{{X}}} \mathord{\left/ {\vphantom {{{{Y }} = {\rm{ }}{{{B}}_{{\rm{max}}}}*{{X}}} {\left( {{{{K}}_{\rm{D}}}{{ + X}}} \right)}}} \right. } {\left( {{{{K}}_{\rm{D}}}{{ + X}}} \right)}}\end{equation*}$$

where *Y* is specific binding, *X* is TFAM concentration, and Bmax is the maximum specific binding. The graph was plotted using Prism (GraphPad Software Inc.). The quantified data were presented as mean ± SEM (standard error of the mean) from three independent experiments. The statistical significance was calculated with a two-tailed unpaired *t* test.

### Fluorescence polarization

The DNA substrates were the same as those in EMSA, but labeled with 6-FAM instead of Cy3. The reaction mixture (20μl) contained 10 mM HEPES pH 8.0, 150–400 mM NaCl, 2 mM DTT, 0.1 mg/ml BSA, 1 nM 6-FAM labeled DNA duplex and TFAM (0–20 μM). The mixture was incubated at RT for 10 min and 15 μl of sample was transferred to a 384-well black microplate. Fluorescence polarization was recorded using a microplate reader (CLARIOstar, 482–16 nm excitation and 530–40 nm emission filters). All experiments were triplicated. *K*_D_ values were calculated as above, and the graph was plotted using Prism.

### EcoRI cleavage assay

A 100-bp substrate was obtained by PCR amplification of a plasmid using the following primers: 5′-CTGAAGCCAGTTACCTTTGAAAAAAG-3′ and 5′-TAATCTGCTGCTTGCAAATAAAAAAAC-3′. DNA2^GG^ was designed to have a single GN_10_G consensus and an EcoRI cleavage site between the two guanines. Both guanines were replaced with adenines in DNA2^AA^. DNA2^GG^: 5′- CTGAAGCCAGTTACCTTTGAAAAAAGAGTTGGTAGCTCTTGATC**C**AG*GAATTC*AA**C**CACCGCTGGTAGCGGTGGTTTTTTTATTTGCAAGCAGCAGATTA-3′; DNA2^AA^: 5′-CTGAAGCCAGTTACCTTTGAAAAAAGAGTTGGTAGCTCTTGATC**T**AG*GAATTC*AA**T**CACCGCTGGTAGCGGTGGTTTTTTTATTTGCAAGCAGCAGATTA-3′. The underlined italic letters indicate the EcoRI cleavage site. Each binding mixture contained buffer (10 mM Tris–HCl pH 7.9, 10 mM MgCl_2_, 100 μg/ml BSA, 50 mM NaCl), 100 nM DNA substrate and 0–50 nM TFAM. The reaction mixture was incubated 30 min on ice. 0.5 unit of EcoRI was then added and incubated for 15 min at 37°C. The reaction was terminated by the addition of an equal volume (20 μl) of 1% SDS, 20 mM EDTA, 300 mM sodium acetate, followed immediately by phenol-extraction. The extracted DNA was ethanol-precipitated overnight. DNA was pelleted and resuspended in loading buffer and loaded on a 4% agarose gel. The gel was stained with SYBR^®^ Gold (Invitrogen) and visualized using a Typhoon 9000. The results were analyzed using ImageQuant (GE Healthcare). The quantified data were presented as mean ± SEM (standard error of the mean) from three independent experiments. The statistical significance was calculated with a two-tailed unpaired *t* test.

## RESULTS

### TFAM preferentially binds to a GN_10_G consensus on DNA substrates

**Figure 1.**
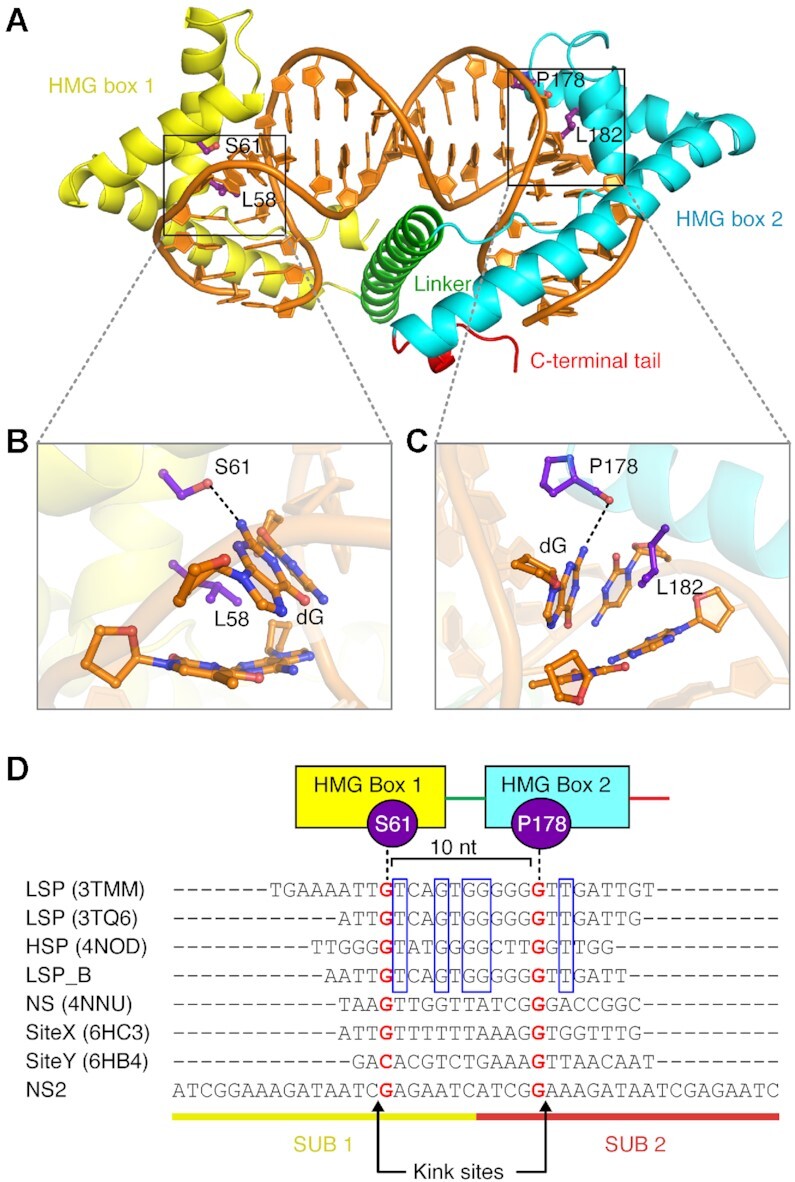
Interactions on GN_10_G consensus. (**A**) Overall structure of TFAM-LSP_B. (**B**) Zoom-in view of the interaction between the guanine and Ser61 at HMG box 1. The dotted line indicates hydrogen bonds. (**C**) Zoom-in view of the interaction between the guanine and Pro178 at HMG box 2. (**D**) Sequence alignment of DNA substrates in all TFAM crystal structures. The consistent interactions observed in all crystal structures between the residues and GN_10_G consensus are marked. Although the distances between Ser61/Pro178 and guanine bases are a little variable, the averaged distances of both interactions are within hydrogen-bonding (3.18 Å for Ser61 and 3.28 Å for Pro178 on models adjusted by PDB-REDO (57)) (Supplementary Table S1). Two guanine bases in GN_10_G are shown as red letters. The blue boxes indicate the aligned nucleotide sequences among promoter sites.

Existing crystal structures of TFAM in complex with promoter sequences (19,20) show that the two HMG boxes, linked by an α-helix, tightly bind the DNA substrate and bend it by intercalating two residues from each of the HMG boxes. However, the structures do not reveal obvious sequence-specific interactions between TFAM and the DNA bases. Although DNA bending, residue intercalations and some hydrophobic interactions by HMG boxes could confer binding specificity (32–34), the fact that crystal structures in complex with non sequence-specific substrates present similar structural features raises the question of which features are involved in sequence-specific recognition (19,20,30). TFAM interacts with promoter sequences mostly through non-sequence specific contacts with the phosphates and riboses of DNA, as well as non sequence-specific hydrogen bonds to the DNA minor groove (Supplementary Figure S1). Although direct base contacts between TFAM and promoter sequences also exist, most of the interactions at LSP and HSP1 are not conserved. Furthermore, most of these interactions, established by Thr78, Arg157, Tyr162, Asn163 and Gln179 of TFAM, consist of hydrogen-bonding to the universal acceptors in the minor groove, and therefore could in principle be established with any DNA base (19,20,30). However, close inspection of both structures of TFAM in complex with its LSP binding site (3TMM and 3TQ6) revealed two guanine-specific interactions: two TFAM residues in each HMG box, Ser61 (in HMG box1) and Pro178 (in HMG box2) specifically hydrogen-bond to two different guanine bases (Figure 1A–C). The Oγ of Ser61 in HMG box 1 interacts with N2 of one guanine base (Figure 1B). Interestingly, another residue, Tyr57, interacts with the minor groove acceptor of the same base. The N2 of a second guanine base is recognized by the main chain carbonyl of Pro178 (Figure 1C).

Because in the reported crystal structures the DNA ends stack to generate a circular structure within one (3TQ6) or two asymmetric units (3TMM), we wondered if the positioning of TFAM in the DNA molecule could be determined by crystal packing. We thus decided to solve an additional structure of TFAM bound to its LSP binding site (TFAM-LSP_B/PDBID: 7LBX) after shifting the sequence by one nucleotide with respect to 3TQ6 (Figure 1D). We were able to obtain data to 2.7 Å (Table 1). Despite the shift, as in other crystal structures of TFAM-DNA substrates (19,20,30) (Supplementary Figure S2), in the TFAM-LSP_B structure, two HMG boxes interact tightly with the DNA duplex via the minor groove and generate two kink sites the intercalations of Leu58 (HMG box1) and Leu182 (HMG box2), inducing a U-turn in the DNA substrate. The α-helix linker connecting the two HMG boxes further enhances the binding of TFAM to the DNA duplex (Figure 1A and Supplementary Figure S2). Despite subtle differences in the sequence, the structure of TFAM-LSP_B revealed the same guanine-specific interactions as the other two TFAM-LSP structures, further highlighting the importance of these interactions and indicating that the TFAM binding site is not modulated by crystal packing. Two additional crystal structures of TFAM in complex with an LSP-like sequence, Site-X and -Y, have been determined (35). Both show interactions consistent with a GN_10_G consensus (Supplementary Figure S3A and B). An additional TFAM structure with the HSP1 binding site published in 2014 displays an identical interaction (30). Furthermore, the same pattern of interactions can be observed in a crystal structure of TFAM bound to a nonspecific sequence (NS) (4NNU, Figure 1D) (30). Except for the GN_10_G consensus, we could not find any sequence conservation among the different sequences found in the crystal structures (Figure 1D). Although promoter sequences display sequence conservation (blue boxes, Figure 1D), no interactions were observed between the conserved bases and TFAM residues in the structures, except for a non-bonded interaction of Ile81 (30), suggesting that these bases did not contribute to specific binding of TFAM.

**Table 1.** Statistics of data collection and structure refinement

Crystal	TFAM-LSP_B	TFAM-NS2
PDB	7LBX	7LBW
Space group	*P* 2_1_ 2_1_ 2	*P* 2_1_ 2_1_ 2
Unit cell		
* a*, *b*, *c* (Å)	113.4, 120.5, 55.2	115, 124.8, 55.2
α, β, γ (°)	90, 90, 90	90, 90, 90
Resolution (Å) ^a^	82.6–2.7 (2.75–2.7)	39.8–2.84 (2.85–2.84)
Wavelength (Å)	1.075	1.1
*R_merge_ (%)* ^a^	4.6 (70.6)	6.1 (63.6)
*I* / σ ^a^	29.4 (2.7)	33.6 (4.0)
CC_1/2_^a^	1.0 (0.82)	1.0 (0.95)
Completeness (%) ^a^	99.7 (99.9)	100 (100)
Multiplicity ^a^	7.7 (8.1)	12.8 (13.1)
Wilson B (Å^2^)	66.5	69.3
**Refinement**		
Resolution (Å) ^a^	29.1–2.7 (2.84–2.7)	39.8–2.84 (2.99–2.84)
No. reflections	21 463	19 442
No. reflections for R_free_	1012	996
*R*_work_ / *R*_free_	0.208/0.242	0.199/0.232
No. atoms		
Overall	5198	5258
Protein	3213	3223
DNA	1788	1767
Ligand	22	45
Water	175	223
Mean *B*-factors (Å^2^)		
Overall	84.7	80.6
Protein	84.1	77.1
DNA	87.2	88.5
Ligand	84.5	73.7
Water	70	70.2
R.M.S. deviations		
Bond lengths (Å)	0.013	0.013
Bond angles (°)	1.4	1.4
Ramachandran		
Favored (%)	98.67	98.15
Allowed (%)	1.33	1.85
Outliers (%)	0	0

^a^Values in parenthesis are of the highest resolution shell.

In summary, all existing structures suggest that TFAM binding appears to be organized around a GN_10_G consensus, where two guanine bases are separated by 10 nucleotides (Figure 1D; Supplementary Figure S1).

### The GN_10_G consensus plays an important role in mitochondrial transcription

**Figure 2.**
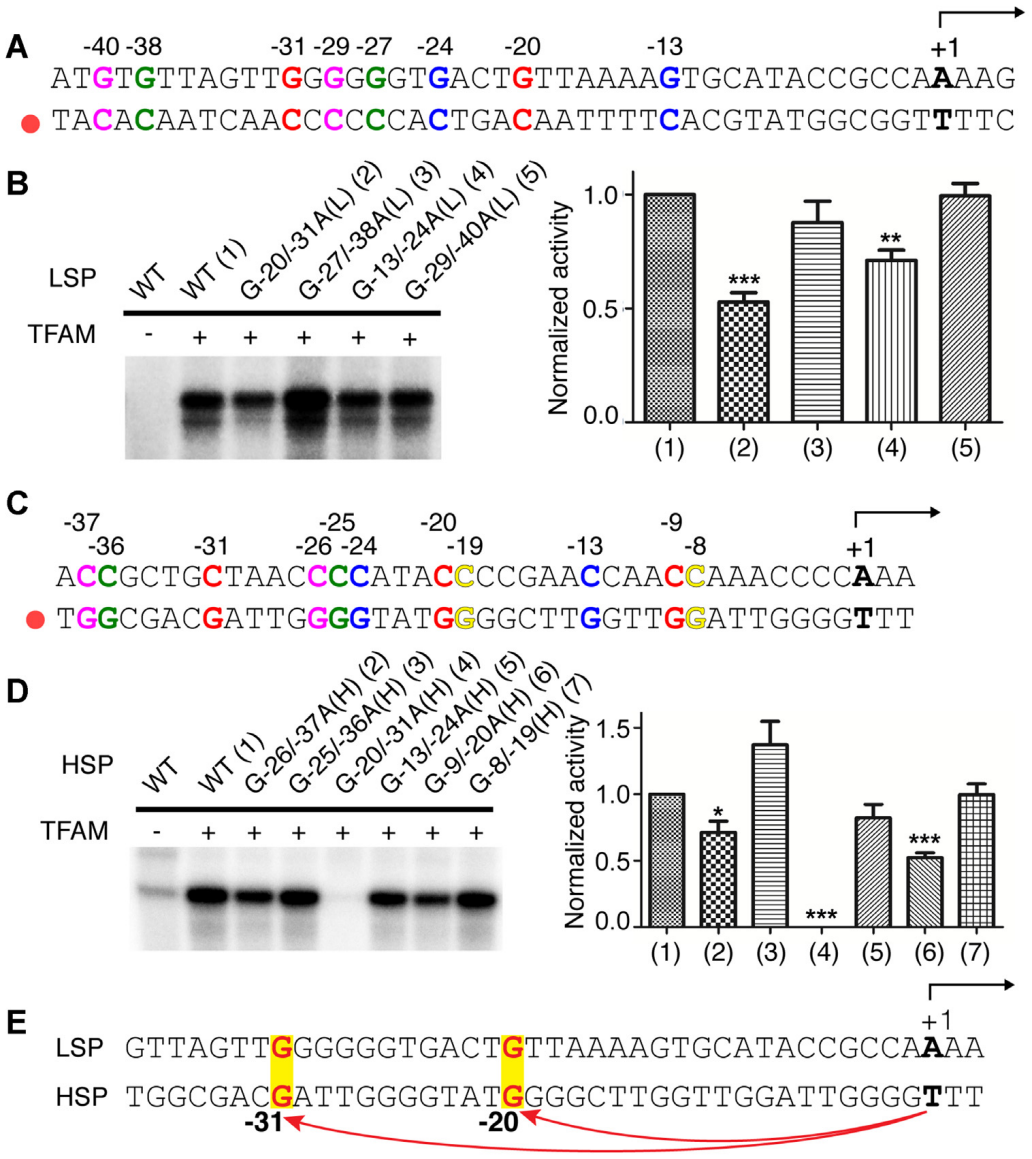
Role of the GN_10_G consensus in transcription. (A, C) Upstream sequences of the LSP (**A**) and HSP1 (**C**) containing the TFAM binding site. Different GN_10_G sites are marked with different colors. Residues are labeled with respect to the transcription start site (marked as +1). L/H indicates the strand where the base is present. The black arrow represents the direction of transcription. The red circle indicates the template strand. (B, D) *In vitro* transcription assay with different GN_10_G substitutions on LSP (**B**) and HSP1 (**D**). The left panel shows the transcription run-off products in the TBE-Urea gel. The right panel shows the quantified result. The error bars are the standard error of the mean (SEM) calculated from three independent experiments. The statistical significance was calculated with a two-tailed unpaired *t* test. *** *P*< 0.001, ** *P*< 0.01, * *P*< 0.05. (**E**) Alignment of LSP and HSP1. The GN_10_G consensus that is relevant for transcriptional initiation is shown in yellow shade and bold red letters.

We then decided to investigate the importance of the GN_10_G consensus for mitochondrial transcription initiation. The crystal structures with the LSP binding site (3TMM and 3TQ6) indicate that the G-20/-31 guanine pair (Figure 2A) should drive TFAM binding (19,20). However, several additional occurrences of a GN_10_G consensus appear around this site. In order to test the importance of each individual guanine pair, we carried out *in vitro* transcription assays after systematically modifying the transcription substrate. Replacement of both guanines in the expected guanine consensus to adenines (G-20/-31A(L), L stands for LSP) resulted in a ∼50% decrease in transcription activity (Figure 2B). Conversely, altering three additional surrounding guanine pairs around the TFAM binding site (G-27/-38A(L), G-13/-24A(L) and G-29/-40(L)) had almost no influence in transcriptional initiation activity (Figure 2B). Replacement of G-13/-24(L) did result in a ∼30% reduction in activity. However, this reduction is likely related to the fact that G-13(L) is located in the binding site for the POLRMT/TFB2M complex (44). Thus, our results confirm that the G-20/-31(L) is important for proper transcriptional activity of TFAM. At the same time, the fact that eliminating the guanine pair does not completely eliminate initiation activity indicates that other factors must be involved in specific recognition of LSP by TFAM. We further investigated the importance of the interaction with the GN_10_G consensus by mutating Ser61 of TFAM to alanine (S61A). Unfortunately, it is not possible to eliminate the hydrogen bond acceptor in Pro178 because the atom involved in the interaction is its main chain carbonyl. S61A led to a decrease of ∼30% in transcription activity (Supplementary Figure S4A). As expected, this reduction is more modest than that observed when mutating the two guanines in the consensus.

We next examined the importance of the GN_10_G consensus for HSP1 transcription initiation. We thus systematically altered six different guanine pairs close to the HSP1 transcription start site (Figure 2C). Among these, mutation of G-20/-31(H) (H stands for HSP1) resulted in an almost complete abrogation of stimulation of initiation activity by TFAM (Figure 2D). Compared to this reduction, alteration of the G-25/-36A(H), G-13/-24A(H), and G-8/-19A(H) pairs did not significantly affect initiation activity. The mutation of G-9/-20(H) led to ∼50% activity reduction. However, this consensus shares G-20(H) with G-20/-31A(H), and ExoIII footprinting studies showed that G-9(H) is located in the binding site for the TFB2M/POLRMT complex (44). Interestingly, alteration of G-26/-37(H) reduced the activity by ∼30%. Although crystal structures have not identified specific interaction between the C-terminal tail of TFAM and DNA, G-26(H) is close to the tail suggesting that it might be involved in a transient interaction. Similar to what was observed at LSP, replacement of Ser61 to Ala also reduced transcription activity by ∼25% (Supplementary Figure S4B). In conclusion, our results indicate that the key GN_10_G pair for HSP1 transcription appears to be G-20/-31A(H).

Interestingly, on both LSP and HSP1, the guanine pair relevant to transcription initiation is placed at an identical distance (G-20/-31) upstream from the transcription start site (Figure 2E). This further supports that a common TFAM binding mechanism drives both LSP and HSP transcription.

The results of our *in vitro* transcription assays allowed us to conclude (i) that TFAM binding to a GN_10_G consensus plays a central role in both LSP and HSP1 transcription initiation, (ii) that each promoter has a single specific consensus that results in productive TFAM binding for transcriptional activation despite the presence of several guanine pairs present around the TFAM binding sites and (iii) that binding to the GN_10_G consensus appears to be more important for HSP1 than for LSP transcription initiation. This is consistent with the fact that TFAM binding to LSP appears to be highly specific (21,22) and might reflect a larger dependence on binding the correct GN_10_G site for initiation at HSP1.

### The GN_10_G consensus contributes to TFAM binding throughout mtDNA

In the context of its mtDNA packaging function, TFAM must interact with multiple sequences throughout the mitochondrial genome. Crystal structures of TFAM bound to non-specific sequences suggest that even in this context TFAM associates with a GN_10_G consensus (30). In order to study whether this consensus can influence nonspecific binding, we decided to study whether the presence of a GN_10_G consensus could affect the DNA binding affinity of TFAM by fluorescence polarization experiments. We designed a 28-bp nonspecific oligonucleotide corresponding to a region of the human mtDNA (6694–6721; within the cytochrome C oxidase subunit I gene), that contains a single GN_10_G consensus (DNA^GG^) and compared it to an identical sequence where this consensus had been eliminated by replacing the two guanines with adenines (DNA^AA^).

**Figure 3.**
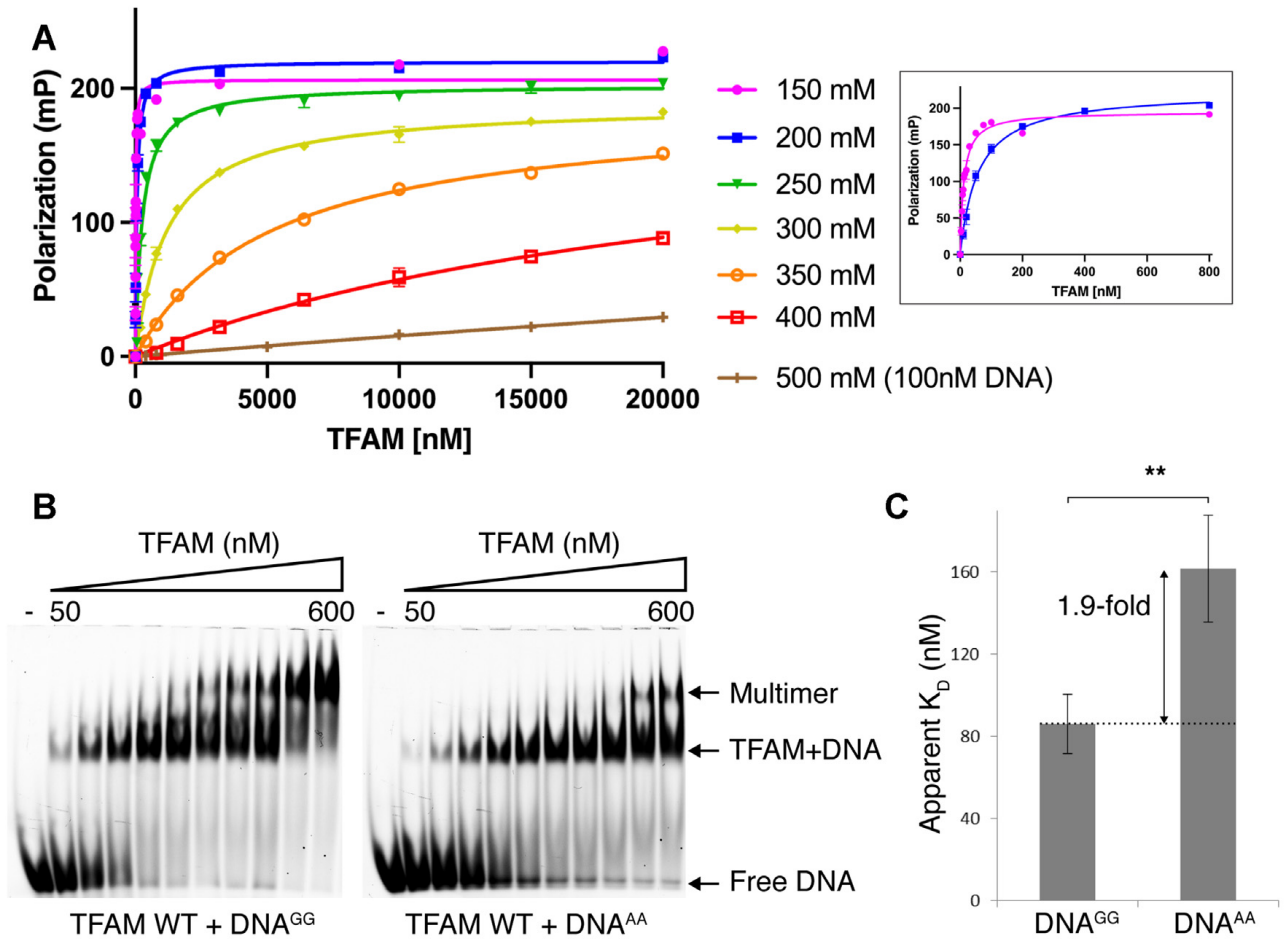
Effect of the GN_10_G consensus on the binding affinity of TFAM (**A**) Fluorescence Polarization assay. The affinity of TFAM to DNA is reduced with increased ionic strength. Assays were conducted with 1 nM DNA by increasing NaCl concentration (150–500 mM). Despite increasing the substrate concentration to 100 nM, the stability of the TFAM/DNA complex in solution was too low to permit a reliable measurement of the *K*_d_. The inset shows binding at lower TFAM concentrations at 150 and 200 mM NaCl. (**B**) Gel shift of DNA^GG^ (left) and DNA^AA^ (right) with TFAM WT. (**C**) Quantification of the results on (B). The arrow indicates the difference in apparent *K*_D_ between DNA^GG^ and DNA^AA^. The error bars are the standard error of the mean (SEM) calculated from three independent experiments. The statistical significance was calculated with a two-tailed unpaired *t* test. ** *P*< 0.01

We attempted to measure the binding affinity of TFAM to DNA using fluorescence polarization. In this assay, both binding affinities between DNA^GG^ and DNA^AA^ were similar in physiological conditions (150–200 mM NaCl) and consistent with previous studies (13.5 ± 0.1 nM for DNA^GG^ and 12.0 ± 1.5 nM for DNA^AA^ at 150 mM NaCl; Supplementary Table S2) (19,29,30,45–48). We hypothesized that under these conditions, the large number of electrostatic interactions established between TFAM and DNA largely drive the binding energetics. We thus decided to conduct binding experiments at higher salt concentrations. However, although binding could be observed even at 500 mM NaCl, above 350 mM the binding affinity was too low to allow us to accurately calculate a binding constant (Figure 3A and Supplementary Table S2). It has been reported that gel-based assays can in some circumstances enhance the stability of a protein-DNA complex (49–52). Indeed, even at 500 mM NaCl, EMSA assays allowed us to observe binding of TFAM to both DNA^GG^ and DNA^AA^ (Figure 3B). We used this approach to calculate an apparent *K*_D_. This revealed a modest but significant decrease in binding affinity to DNA^AA^ compared to DNA^GG^ (Figure 3C and Table 2). Interestingly, increasing TFAM concentration led to supershifts in both DNA^GG^ and DNA^AA^, and the additional shift is strongly affected by the presence of a GN_10_G consensus in the sequence (Figure 3B).

**Table 2.** Binding affinity (apparent *K*_D_) of TFAM with DNA substrates

Substrate	Apparent K_D_ (nM)
**DNA^GG^**	86.1 ± 14.3
**DNA^AA^**	161.7 ± 26.0

**Figure 4.**
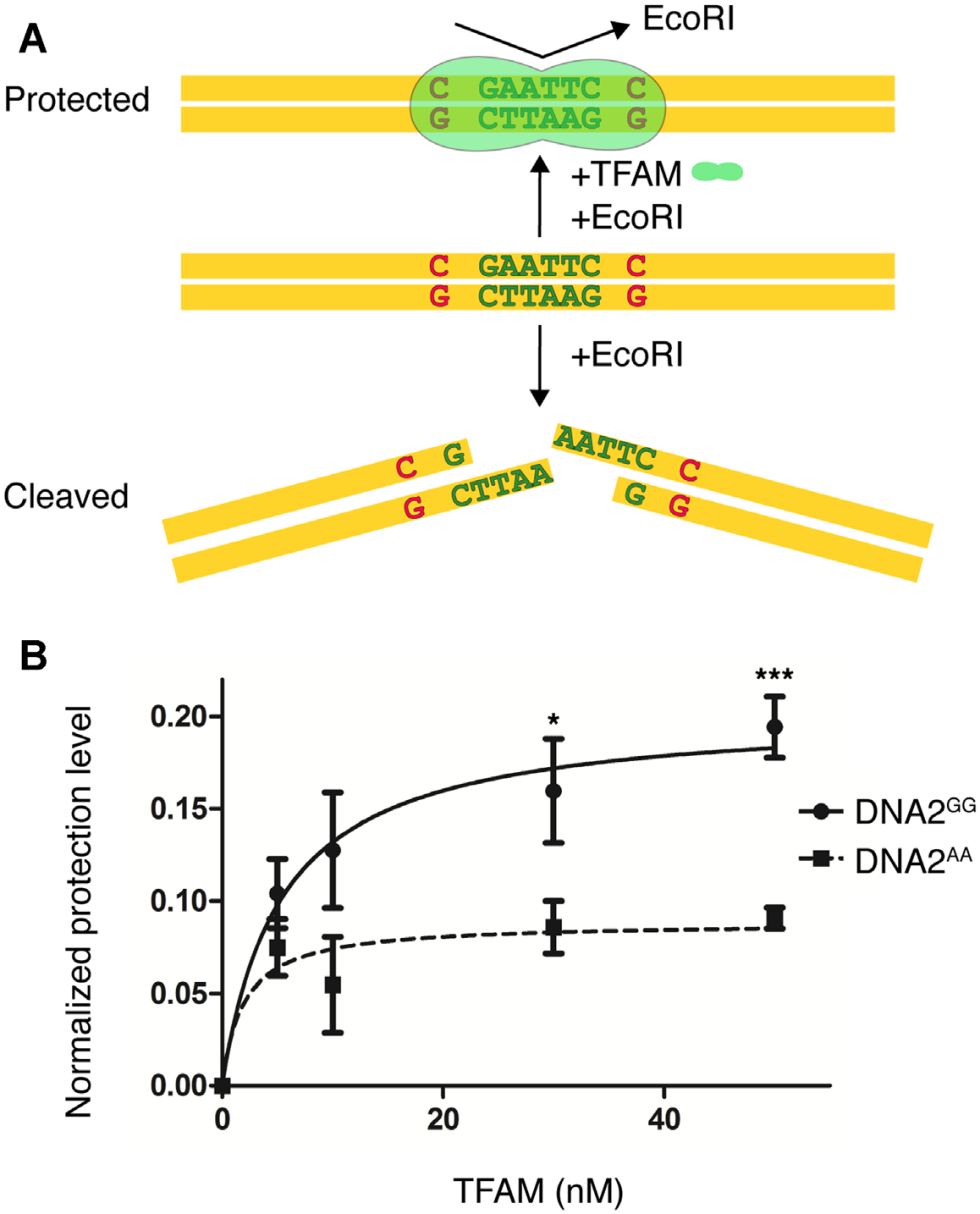
EcoRI cleavage assay. (**A**) Experimental design. The GN_10_G site is indicated with red letters. The green letters indicate the EcoRI cleavage site. (**B**) Quantification of substrate protection by TFAM on DNA2^GG^ and DNA2^AA^. The error bars are the standard error of the mean (SEM) calculated from three independent experiments. The statistical significance was calculated with a two-tailed unpaired *t* test. *** *P*< 0.001, * *P*< 0.05.

Although EMSA assays showed a preference for binding the consensus, they only revealed a small difference in affinity. Thus, to further confirm the role of the GN_10_G consensus, we developed an additional binding assay. We utilized a 100-bp substrate engineered to contain a single EcoRI site flanked by a GN_10_G consensus (Figure 4A). We hypothesized that if the consensus leads to preferential TFAM binding, the presence of TFAM will protected the substrate from EcoRI cleavage. We then compared the effect of TFAM binding on this substrate (DNA2^GG^) and DNA2^AA^, where the consensus was eliminated by replacing both guanines with adenines. As expected, a high TFAM/DNA ratio can mostly prevent cleavage in both substrates (Supplementary Figure S5). Lower TFAM/DNA ratios showed a statistically significant difference in the protection of the two substrates (Figure 4B), further indicating that TFAM prefers to bind to a GN_10_G consensus.

### Bridging of two DNA molecules by TFAM

**Figure 5.**
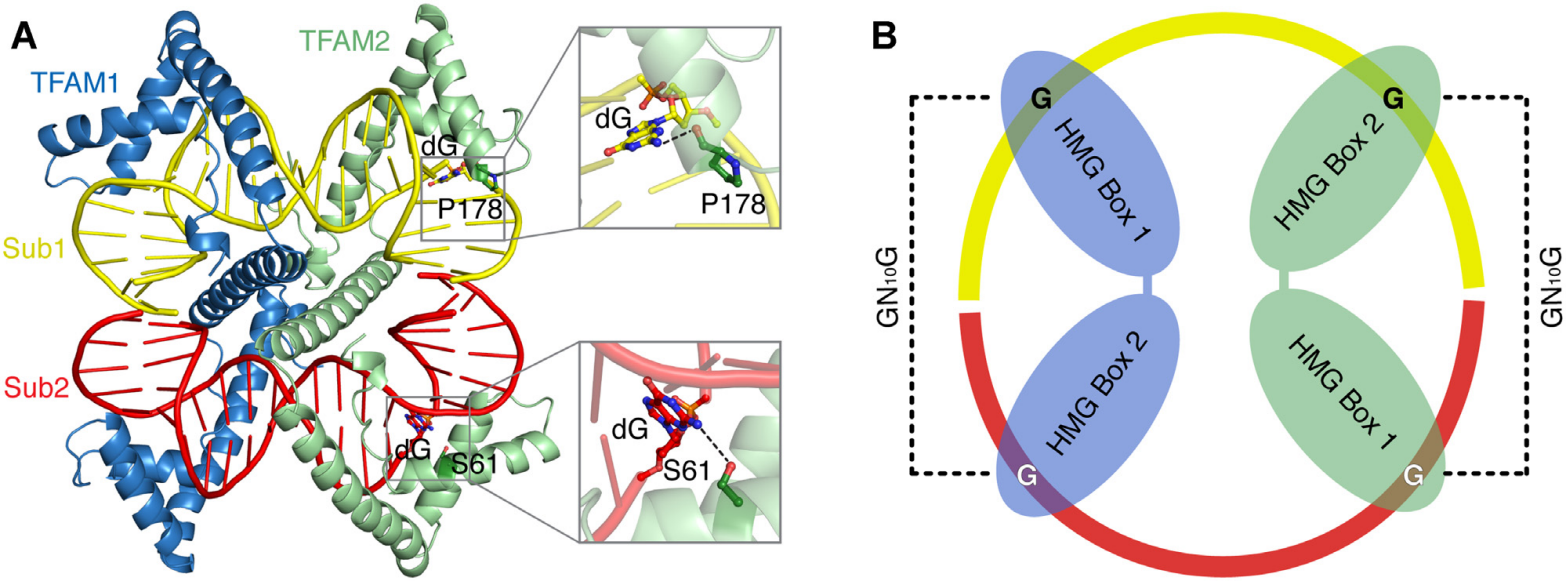
Binding of TFAM to two DNA ends. (**A**) Overall structure of the asymmetric unit of TFAM-NS2. (**B**) Diagram of TFAM bound to a GN_10_G consensus that spans two DNA molecules. The GN_10_G guanine bases are shown as black or white letters, and the consensus is also labeled with dashed lines.

In order to further investigate whether the GN_10_G consensus could drive TFAM binding to DNA, we decided to crystallize TFAM in complex with a 22-mer nonspecific sequence (NS2) containing a single GN_10_G consensus where all other nucleotides were randomized with respect to the sequence used for our LSP structure. We obtained crystals that diffracted to 2.84 Å and we solved the crystal structure by molecular replacement using the TFAM-LSP (3TQ6) structure as a search model. Strikingly, the TFAM-NS2 structure showed a unique arrangement. All TFAM-DNA structures determined to date have revealed that a molecule of TFAM interacts with a molecule of DNA (1:1 ratio of TFAM to DNA) (19,20,30,35). However, in the TFAM-NS2 structure, the binding site of TFAM was composed of two halves of adjacent DNA molecules (Figures 1D and 5A). HMG box 1 of a TFAM molecule interacts with half of a DNA molecule, while HMG box 2 binds to a different DNA molecule (see scheme on Figure 5B). Both DNA ends form stable base stacking (Supplementary Figure S6). In order to confirm that TFAM is indeed bridging two DNA molecules, we further investigated whether electron density corresponding to a phosphate could be observed between the two DNA ends. Since our synthesized DNA substrates lacked a 5′ phosphate, the absence of electron density for a phosphate indicated the location of the DNA ends. Simulated annealing omit maps failed to reveal electron density corresponding to a phosphate. Moreover, we built a phosphate residue to join the two DNA ends and carried out occupancy refinement to determine the occupancy of the extra phosphate. This confirmed that all TFAM molecules in the crystal are bridging two DNA molecules and that this is the only arrangement present in the structure.

Intriguingly, the TFAM-NS2 structure was also bound a GN_10_G consensus, although in this case the consensus was formed between two adjacent DNA molecules (Figure 5B). Similar DNA bridging was observed in the crystal structure of the yeast homolog of TFAM, Abf2p (53). Although Abf2p does not have an α-helix linker between both HMG domains, the structural conformations of HMG domains on DNA are consistent with those of human TFAM: two intercalations by each HMG box induce a U-turn-shaped DNA form. The fact that this binding mode is observed for both TFAM and Abf2p suggests that it reflects a property of both proteins.

## DISCUSSION

TFAM is a key mitochondrial protein involved in both mitochondrial transcription initiation by recognizing specific promoters and mtDNA packaging by binding to any non-sequence specific sites. Its binding to DNA substrates is mostly mediated by electrostatic interactions via its tandem HMG boxes. In this study, we identified two TFAM residues, Ser61 in HMG box1 and Pro178 in HMG box2, that form specific hydrogen bonds with N2 of two guanine bases separated by 10 nucleotides (GN_10_G consensus). Strikingly, binding to a GN_10_G consensus has been observed in all determined crystal structures of a TFAM complex, regardless of whether they involve promoter or non-promoter DNA substrates (Figure 1D) (19,20,30,35). Our biochemical studies support the importance of the GN_10_G consensus. Importantly, the presence of a GN_10_G consensus at the appropriate distance from the promoter appears essential to facilitate transcriptional initiation. Moreover, our results suggest that binding to a GN_10_G consensus also takes place in the context of the mtDNA packaging function of TFAM. The identification of the GN_10_G consensus provides insight into the mechanisms of sequence recognition by TFAM. Importantly, human mtDNA is guanine-rich, and thus this binding strategy appears to be appropriate to facilitate binding of TFAM throughout the genome. However, two specific hydrogen bonds are clearly insufficient to drive specific binding, suggesting that other residues interacting with promoter sequences must contribute to sequence specificity. Moreover, TFAM might also take advantage of additional mechanisms like shape readout (33,54,55) to recognize sequence-dependent conformations in the DNA duplex.

The guanine-specific interactions in GN_10_G occurs via two residues in each HMG box (Ser61 in HMG box 1 and Pro178 in HMG box2). In addition to Ser61, the binding to the guanine at HMG box1 might be further enhanced by Tyr57, which might be consistent with higher binding affinity of HMG box1 compared to HMB box2 (19,30,45).

Our results suggest that both LSP and HSP1 contain a single specific GN_10_G consensus that TFAM needs to bind for efficient transcription initiation (Figure 2). The absence of these guanine pairs likely results in inaccurate positioning of TFAM, leading to incorrect recruitment of POLRMT. Furthermore, it has been shown that for both promoters, it is essential that the C-terminal tail of TFAM is located in proximity to the transcription start site (16,19,30,31). Although the exact function of the tail on transcription initiation is not yet understood, it is likely that association with alternative GN_10_G pairs on promoters would incorrectly position the C-terminal tail, leading to less effective or no transcription initiation. Interestingly, the guanine pairs are located at the same relative distance to the transcription start site (−20 and −31) in both promoters (Figure 2E), consistent with the arrangement observed in crystal structures of the transcription initiation machinery (15). Interestingly, recognition of the guanine pairs seems to be more important for HSP1 than for LSP transcription initiation (Figure 2B and D). Since no additional base-specific interactions are observed in the LSP structures, this suggests that sequence-dependent conformations of the DNA duplex might facilitate the formation of the additional base contacts that are only observed in LSP structures. Thus, shape readout might be more relevant to recognition of the LSP promoter.

The recognition of the GN_10_G consensus appears to be related to DNA bending. Two kink sites are located adjacent to both guanine bases in the consensus (X**↓G**N_10_**G↓**X) (Figure 1D). The guanine-specific interactions in GN_10_G with TFAM appear to fix and stabilize the bound DNA substrate, which might contribute to more efficient insertion of Leu58 and Leu185, generating the two kinks.

Ngo, HB *et al.* showed that TFAM can dimerize via a helix-helix interaction between each HMG box 1, and that dimerization is required for DNA compaction (30). This behavior might be related to the supershifts observed in our EMSA experiments, which might represent dimerized TFAM species. It is interesting to note that these complexes were formed much more readily in the presence of a GN_10_G consensus, highlighting its potential importance for the mtDNA packaging function of TFAM. Furthermore, the observation that higher TFAM concentrations result in more supershifts (Figure 3B) indicates that the oligomerization of TFAM is concentration-dependent, which was further supported by Cuppari *et al.* (35).

The TFAM-NS2 crystal structure captured a unique complex in which TFAM is bridging two separate DNA molecules. Since nothing in our experimental setup prevented TFAM from binding a single DNA molecule, under our experimental conditions DNA bridging appears to be preferred, although the reason for this preference is unclear. A similar structure has also been observed for Abf2p, the yeast TFAM homolog (53), indicating that the ability to bridge DNA ends is common to both proteins. Importantly, Abf2p plays no role in transcriptional initiation, perhaps suggesting that this ability might be relevant to the role of these proteins in mtDNA packaging. Furthermore, the NS2 sequence contains possible A-tracts (AAA or ATAAT) which play a key role in the bridging arrangement of Abf2p structures (53). This might indicate that the partial A-tract in NS2 might also play a role in the unique binding arrangement observed in the TFAM-NS2 structure. Alternatively, these structures might be relevant to a potential role of these proteins stabilizing broken DNA ends. Although no evidence currently exist supporting such a role, HMGB1, a tandem HMG box-containing protein similar to TFAM has been suggested to play a role in double-strand break (DSB) repair (56).

In conclusion, our biochemical and structural studies provide insight into the mechanism of DNA binding by TFAM. We have demonstrated that TFAM binding involves a small consensus, and that binding to this consensus appears to be important for TFAM binding and proper positioning of TFAM at the promoters to facilitate efficient transcriptional initiation.

## DATA AVAILABILITY

The crystal structures (TFAM-LSP_B and TFAM-NS2) are deposited to the Protein Data Bank with accession codes 7LBX and 7LBW, respectively.

## Supplementary Material

gkab1230_Supplemental_File

## Notes

Present address: Woo Suk Choi, Laboratory of Molecular Biology, NIDDK, National Institutes of Health, Bethesda, MD 20892, USA.
