# Supplementary material for: A minimal motif for sequence recognition by mitochondrial transcription factor A (TFAM)
Source: Nucleic Acids Res. 2021 Dec 20;50(1):322–32. doi: 10.1093/nar/gkab1230 (PMC8754647; doi:10.1093/nar/gkab1230)
Supplement: gkab1230_Supplemental_File [file gkab1230_supplemental_file.pdf]

SUPPLEMENTARY DATA

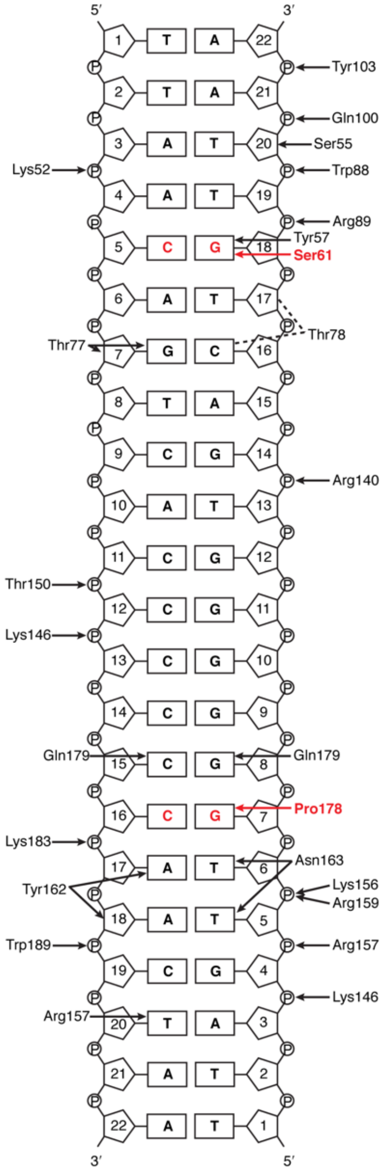

Supplementary Figure S1. Diagram of the interactions between TFAM and LSP. The protein-DNA interactions in TFAM-LSP_B were analyzed using NUCPLOT (1). Hydrogen-bonding and van der Waals interactions are indicated by arrows and dotted lines, respectively. All labelled interactions are under 3.5 Å. The guanine-specific interactions are labeled in red.

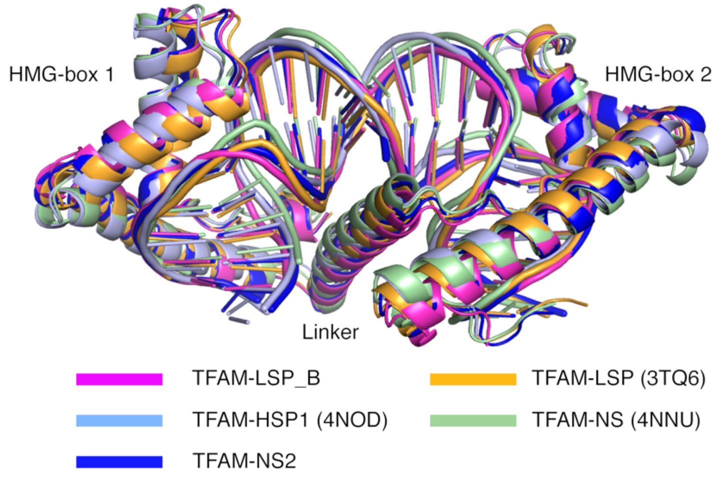

Supplementary Figure S2. Structural overlay of TFAM-LSP_B and TFAM-NS2 with previously reported TFAM crystal structures.
TFAM-LSP_B (pink) and TFAM-NS2 (dark blue) are superimposed with TFAM-LSP (3TQ6), TFAM-HSP1 (4NOD), TFAM-NS (4NNU) (2,3) and TFAM-NS2.

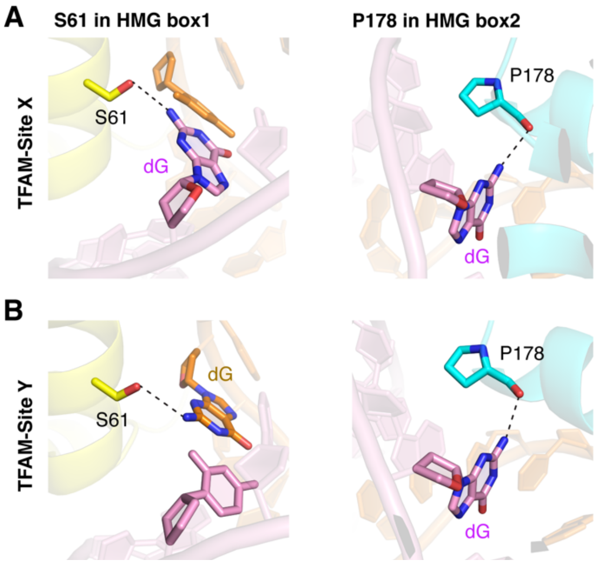

Supplementary Figure S3. $GN_{10}G$ interactions on Site-X and -Y structures

(A) $GN_{10}G$ contact on Site-X by TFAM (PDB ID: 6HC3).

(B) $GN_{10}G$ contact on Site-Y by TFAM (PDB ID: 6HB4). The protein is colored as in Figure 1A. Each strand in the DNA substrate is colored differently (orange and pink). In the TFAM-Site-Y structure, one of the guanines in the $GN_{10}G$ was located on the opposite strand. Yet, both Ser61 and Pro178 interact with N2 of guanine bases from each strand, leading to a pattern of interactions consistent with other structures.

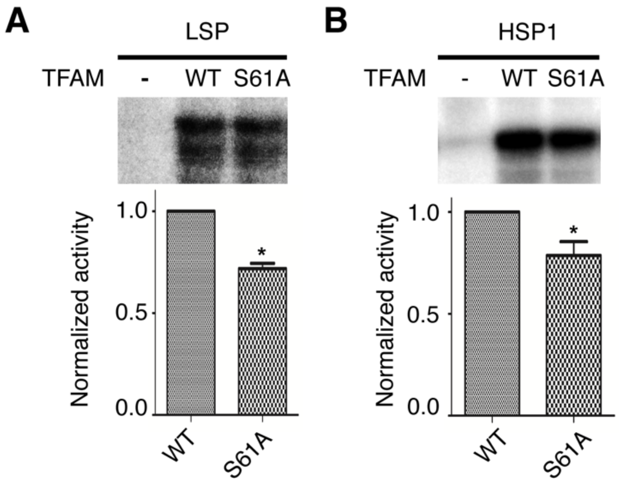

Supplementary Figure S4. Reduction of transcriptional activity resulting from mutation of S61.

(A) *In vitro* transcription on LSP with TFAM S61A.

(B) *In vitro* transcription on HSP1 with TFAM S61A. The transcription run-off products are shown in TBE-Urea gel. The quantified results are shown in the bar graph below. The error bars are the standard error of the mean (SEM) calculated from three independent experiments. The statistical significance was calculated with a two-tailed unpaired t test. * $P<0.05$.

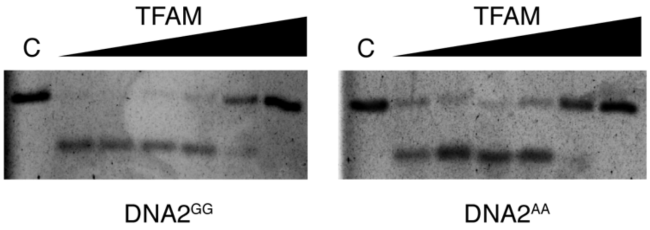

Supplementary Figure S5. High concentrations of TFAM protect DNA substrates regardless of the presence of a $GN_{10}G$ consensus.
C, control. The molar ratio of TFAM to DNA was 0:1, 0.2:1, 0.5:1, 1:1, 3:1, 5:1 (left to right).

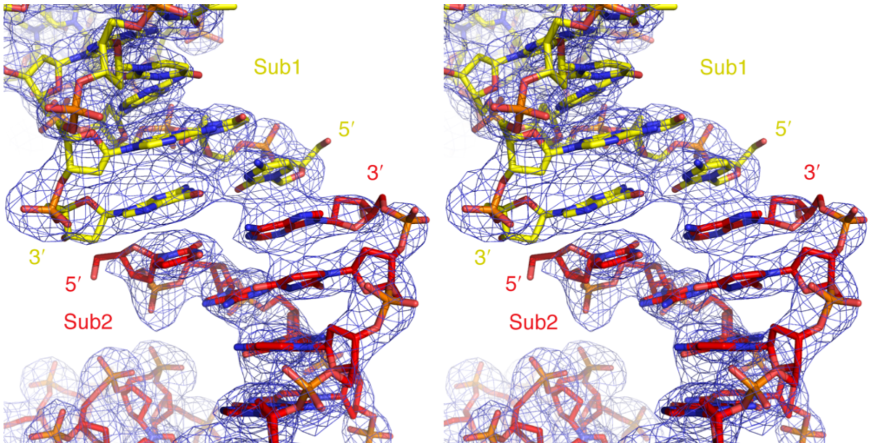

Supplementary Figure S6. Stereo view of base stacking between two DNA ends in TFAM-NS2.

The DNA substrates are colored as in Figure 6. The 2Fo-Fc map is contoured at 1.3 σ.

Supplementary Table S1. Distance between Ser61/Pro178 and the guanine bases in all existing crystal structures.

Some of the contacts not within hydrogen-bonding distances were adjusted by PDB-REDO (4).

Crystal structures	S61 (Å)	P178 (Å)
**3TMM (LSP)**	2.91	3.29
**3TQ6 (LSP)**	3.10	3.04
**4NOD (HSP1)**	3.59 (3.42: PDB-REDO)	3.61 (3.40: PDB-REDO)
**4NNU (NS)**	3.13	3.95 (3.40: PDB-REDO)
**6HC3 (SiteX)**	2.97	3.28
**6HB4 (SiteY)**	3.38	3.16
**LSP_B**	2.95	3.41
**NS2**	3.71 (3.63: PDB-REDO)	3.31

Supplementary Table S2. Dissociation constant measured by Fluorescence Polarization.

The statistical significance was calculated with a two-tailed unpaired t-test. The differences between binding affinities for DNA^{GG} and DNA^{AA} in all NaCl concentrations were not significant.

NaCl	DNA^{GG} (nM)	DNA^{AA} (nM)
150 mM	13.5 ± 0.1	12.0 ± 1.5
200 mM	55.5 ± 6.0	79.0 ± 1.4
250 mM	259.2 ± 48.2	286.9 ± 15.7
300 mM	1209 ± 113	876.9 ± 38.5
350 mM	5179 ± 502	3853 ± 425

1. Luscombe, N.M., Laskowski, R.A. and Thornton, J.M. (1997) NUCPLOT: a program to generate schematic diagrams of protein-nucleic acid interactions. *Nucleic acids research*, **25**, 4940-4945.
2. Rubio-Cosials, A., Sidow, J.F., Jimenez-Menendez, N., Fernandez-Millan, P., Montoya, J., Jacobs, H.T., Coll, M., Bernado, P. and Sola, M. (2011) Human mitochondrial transcription factor A induces a U-turn structure in the light strand promoter. *Nature structural & molecular biology*, **18**, 1281-1289.
3. Ngo, H.B., Lovely, G.A., Phillips, R. and Chan, D.C. (2014) Distinct structural features of TFAM drive mitochondrial DNA packaging versus transcriptional activation. *Nat Commun*, **5**, 3077.
4. Joosten, R.P., Long, F., Murshudov, G.N. and Perrakis, A. (2014) The PDB_REDO server for macromolecular structure model optimization. *IUCrJ*, **1**, 213-220.
